# Continuous or Batch Solid-Liquid Extraction of Antioxidant Compounds from Seeds of *Sterculia apetala* Plant and Kinetic Release Study

**DOI:** 10.3390/molecules23071759

**Published:** 2018-07-18

**Authors:** Federica Mosca, Gádor Indra Hidalgo, Juliana Villasante, María Pilar Almajano

**Affiliations:** 1 Polytechnic of Milan, Department of Chemistry, Materials and Chemical Engineering “Giulio Natta” (CMIC), Piazza Leonardo da Vinci, 32, 20133 Milan, Italy; moscafede@yahoo.it; 2 Chemical Engineering Department, Universitat Politècnica de Catalunya, Av. Diagonal 647, 08028 Barcelona, Spain; chemicontact@gmail.com (G.I.H.); julianavillasante@gmail.com (J.V.)

**Keywords:** continuous extraction, kinetic diffusion, natural antioxidant, *Sterculia apetala*, fatty acids

## Abstract

This work has been intended to investigate the antioxidant properties of compounds extracted from seeds of *Sterculia apetala* (a plant from Central America) in order to add further results to the relatively poor existing literature on the beneficial properties of this plant. Different extraction methodologies were used such as batch or continuous extraction conditions using water or ethanol 50% as solvents. The kinetic study has allowed estimation of the effective diffusion coefficients in a continuous solid-liquid extraction, highlighting the strict dependence of the diffusion rate and temperature and kind of solvent, showing the highest diffusion rate with ethanol 50% at 60 °C. The comparison between different techniques and two solvents led to the selection of water as the best extraction solvent while batch mechanically-agitated extraction was the most efficient mode, with the benefits of use of an environmental-friendly solvent and reduction in process time to achieve a high amount of extracted phenolic compounds. The analysis techniques used were ABTS and Folin-Ciocalteau methods to investigate the antioxidant activity and quantify the Total Phenolic Content (TPC) respectively. Also, different fatty acids were extracted from *Sterculia apetala* seeds and analysed by Gas Chromatography in order to quantify other interesting chemical species besides antioxidants.

## 1. Introduction

Plants have always been consumed by mankind for their nutritional properties and medicinal effects due to the presence of the wide variety of secondary metabolites: terpenes and terpenoids (around 25,000), alkaloids (around 12,000) and phenolic compounds (around 8000) [1]. These bioactive compounds have attracted interest and scientific research has focused on their extraction from natural sources and application to cure diseases, improve health and increase value of nutraceuticals and cosmetics, especially phenolic and flavonoid compounds, due to their antioxidant activity [2]. Antioxidant compounds are not only used as an active principle of certain drugs but their use has also been extended to the food production industry. Many food ingredients contain unsaturated fatty acids which are quite susceptible to deterioration by oxidation and the addition of selected antioxidants can be a valuable solution to this problem [3]. Their protective effect causes a huge delay in the degradation processes taking place after a certain period of storage, prolonging the conservation of quality and dramatically increasing the shelf-life of foods. Synthetic phenolic antioxidants such as butylated hydroxyanisole (BHA) or butylated hydroxytoluene (BHT) effectively inhibit oxidation and are used as food additives but the greater emphasis on consuming foods prepared with only natural ingredients, with familiar names and perceived as healthy, has driven food producers to research natural additives with similar properties to synthetic ones, for a better acceptance by the consumer [3]. The extraction of a solute from a solid matrix involves a three-stage process: desorption of the solutes and solubilisation in the solvent, internal diffusion through the solid phase towards the surface and external solute diffusion in the liquid volume [4]. Extraction can be performed using fresh/dried and ground/whole samples. Usually, dried powdered samples are preferred to fresh whole ones because the lower particle size allows a high surface contact between samples and extraction solvent (favouring the mass transfer of solutes) [4]. The solvent selection is based on the desired quantity of compounds to be extracted, the solvent selectivity with respect to the target compounds, the ease of handling, the potential for health hazards and the cost [4]. Pure ethanol as solvent shows a lower polarity than water and for this reason, aqueous solutions of ethanol are generally preferred to extract polyphenolic compounds (molecules characterized by medium to high polarity, due to the presence of hydroxyl groups) [5]. Mass transport laws rule the extraction process and the final content of antioxidants is mainly dependent on: solvent type and extraction ability, selectivity towards specific molecules, particle size of solid matrix, temperature, extraction conditions (batch or continuous) and flow speed [6]. Batch extraction with stirring means constantly mixing the solvent and the sample to achieve turbulent conditions; concentration gradients are smaller thanks to a higher homogeneity of the extract concentration in the volume and mass transfer is positively favoured by the chaotic movement of vortexes. The transfer process ends when an equilibrium concentration is achieved in the bulk. In continuous extraction, the fresh solvent flow provides high concentration gradients at the interfacial layer between the solid and liquid phase which favours molecular diffusion from the solid surface to the bulk. The process ends when all the extractable compounds are removed from the sample [7]. A temperature increase enhances the solubility of the compounds, matrix-analyte interactions are more easily broken and a higher diffusion rate is achieved following the Einstein equation (Equation (1)) [8]:

(1) $$D=\frac{BT}{6\pi \mu R}$$

where *B* is the Boltzmann constant *µ* is the solvent viscosity and *R* the average particle size. Also, viscosity and surface tension of solvent are reduced and it can penetrate more efficiently into the solid, facilitating the extraction and also reducing the volume of solvent needed [9]. The extraction yield depends on the solvent polarity, temperature, extraction time and composition of the sample [10]. High temperatures modify the solvent properties of water, lowering its polarity and remarkably increasing the solubility of less polar compounds [9]. Maceration is a batch solvent extraction with the achievement of an equilibrium concentration of the extract in the solvent. Continuous solid-liquid extraction is an alternative extraction method. The concentration in the liquid phase versus time data have been usually measured and fitted to theoretical models under appropriate hypotheses to yield values of the diffusion kinetic parameters. Factors such as temperature and flow rate influence the extraction yield and the extraction kinetics of a continuous extraction process (where the solvent flows continuously, “dragging” the polyphenols from the matrix) [11]. Different extraction modes or solvents affect the extraction of bioactive compounds from natural samples (in this work, seeds of *Sterculia apetala*). Sterculia is a genus of a plant originally growing in Central and South America which belongs to the Malvaceae family and presents more than 243 genera and 4225 species distributed worldwide (except in very cold regions) but mainly in South America [12]. Seeds are gathered by local people and consumed as food (boiled, roasted and in some areas used for chocolate flavouring) or used for extracting an oil with various medicinal and pharmaceutical uses. Different studies have investigated the antioxidant, antimicrobial and anti-inflammatory properties of several species of Sterculia with the isolation of flavonoids, alkaloids, phenolic compounds and fatty acids. The authors’ results confirmed the presence of bioactive phytochemicals depending on the kind of sample and part of the plant (bark, roots or seeds) [12]. *Sterculia striata* was studied by Costa et al. who investigated the TPC content and activity of *Sterculia striata* bark obtaining good results and Fontoura et al. studied *Sterculia apetala* bark samples finding related antioxidant properties [12,13]. S. Herrera-Meza et al. investigated the fatty acid composition of the seeds reporting good extraction yield of cyclopropenoic acids, sterculic acid and malvalic acid [14]. This experimental work is focused on the investigation of the antioxidant properties of *Sterculia apetala* and on the extraction of polyphenolic compounds from the seeds with the use of different extraction methods.

## 2. Results and Discussion

### 2.1. Continuous Solid-Liquid Extraction

**Figure 1:**
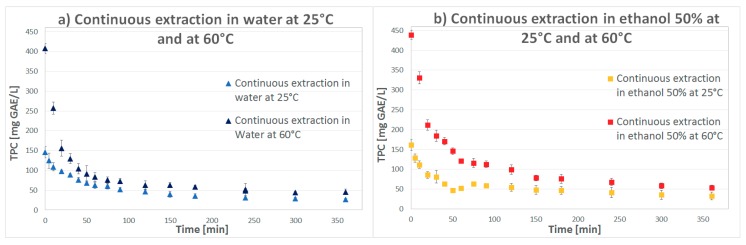
Total phenolic compound concentrations in continuous solvent extraction from seeds using (**a**) water (**b**) ethanol 50%.

**Figure 2:**
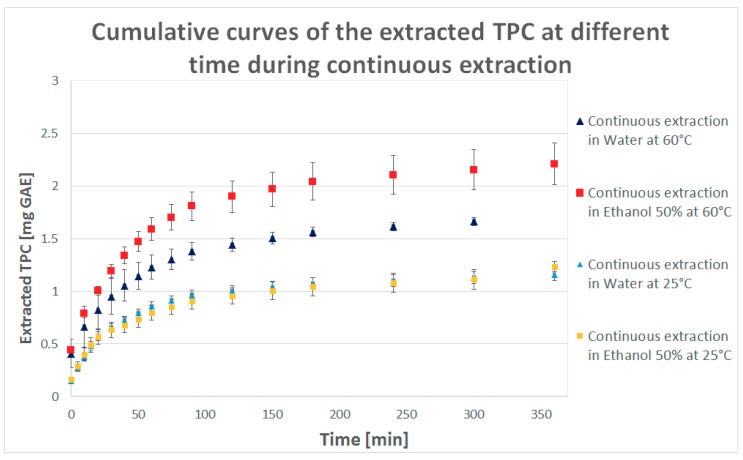
Cumulative curve of the extracted Total Phenolic Content (TPC) at different time during the continuous solid-liquid extraction.

The results are presented as TPC concentration versus time and the cumulative curve of the extracted TPC amount of the collected samples at different time in Figure 1 and Figure 2.

In Table 1, a direct comparison between the use of water or ethanol 50% at different values of temperature is provided: antioxidants are well dissolved and rapidly extracted at the initial “contact” between fresh solvent and sample. The characteristic kinetic extraction curve, after a rapid initial decrease (Figure 1 and Figure 2), reaches a flat trend once the total yield of extractable phenolic compounds was almost achieved, meaning that the biggest part of the antioxidant content was already extracted.

The variation in solvent and temperature conditions show deep differences with respect to the process results: for a lower value of temperature (25 °C) there is no big difference in TPC concentration obtained with the use of water or ethanol 50% as solvent (water provides slightly higher results) while for a higher value of temperature (60 °C) both the solvents lead to a very good improvement on the extraction power (Table 1).

Ethanol 50% presents a lower solvating power at 25 °C than water but increasing the temperature (for which solvent viscosity decreases and dissolution of specific solutes is enhanced), mass transfer is improved [15]: a higher amount of TPC can be extracted, also higher than using water. The antioxidant activity (ABTS) of the collected sample is higher for the extraction at 60 °C with both the solvents (the reported results are those which present the highest values, for samples collected after 10 min) and are higher than the results obtained from samples of Sterculia scaphigera (39.53 ± 4.53 µmol/g) in a previous study used by Sha Li et al. for comparing the antioxidant properties of 223 plants [16].

### 2.2. Estimate of the Effective Diffusion Coefficients

**Figure 3:**
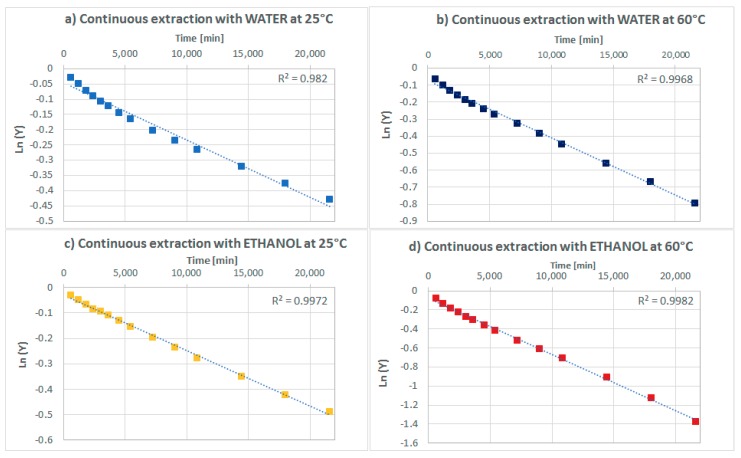
Unextracted fraction of polyphenols against time in a solid-liquid continuous extraction in (**a**) water at 25 °C; (**b**) water at 60 °C; (**c**) ethanol 50% at 25 °C and (**d**) ethanol 50% at 60 °C.

Regarding to the estimate of the effective diffusion coefficient, the construction of the straight line of the logarithm of the unextracted fraction *Y* (Equation (6)) against time allows determination of the value of the slope and, consequently, of the effective coefficient. The shown values of extracted polyphenols are measured at different times for a total extraction time of 6 h. From the experimental data, it has been possible to determine the fraction of unextracted polyphenols (*Y*) in the cases of continuous extraction in water or in ethanol 50% at 25 °C or 60 °C. The plot of the logarithmic *Y* versus time is a straight line where the slope is directly dependent on the value of the effective diffusion coefficient as shown in Figure 3.

Referring to the linearized Fick’s second law solution (Equation (7)), the slope of the straight line (Equation (2)) corresponds to:

(2) $$\mathrm{Slope}=-\frac{\pi^{2}D_{eff}}{r^{2}}$$

where *r* is the radius of the particle (m).

The values of the effective diffusion coefficients are presented in Table 2.

The values of the effective diffusion coefficient are comparable to those found in the literature for the extraction of polyphenols from grape seeds (Pinelo et al., 2006 [17], Sanchéz Guerrero et al., 2008 [11], Mantell et al., 2002 [15]). The increase in temperature decreases the solvent viscosity speeding up the mass transfer rate inside the seed solid matrix. The overall result is that the rate of the extraction process increases with temperature [15] as established by the Einstein equation (Equation (1)) [17]. In the case of a continuous solid-liquid extraction at high temperature (60 °C) ethanol 50% is the best solvent, providing the highest diffusion rate and the highest number of extracted polyphenols.

**Figure 4:**
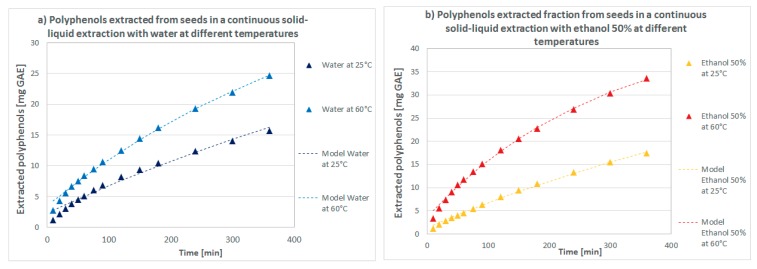
Total polyphenols extracted from *Sterculia apetala* seeds in a solid-liquid continuous extraction in (**a**) water and (**b**) ethanol 50% at 25 °C and 60 °C.

As shown in Figure 4, the used model (Equation (8)) is able to well predict the experimental data in all the cases. Only for the extraction with water, at both values of temperature, a slight deviation is present (for the model fitting the data of the extraction with water at 25 °C). The model slightly overestimates the diffusion rate in the first extraction stage probably due to a different rate of mass transfer during the first period of the extraction [11] which occurs at a slightly lower rate than the second one (from 20 min until 6 h) but this takes place only in the case of water. This could be indicative of the lower solvating power of water compared to ethanol 50% in conditions of continuous solid-liquid extraction at both low and high temperature (25 °C and 60 °C), in contrast to the behaviour found in batch extraction conditions.

### 2.3. TPC Obtained from a Continuous or Batch Solvent Extraction at Different Temperatures

A comparison between batch and continuous extraction is shown below (Table 3). The same solvents (water and ethanol 50%) and the same temperatures (25 °C and 60 °C) but with different extraction periods and particle size of the sample allow interesting considerations on the advantages and disadvantages of the tested techniques.

Considering the differences between the behaviour of the two solvents in the batch extraction, water provided better results at room temperature but a temperature increase seemed to be not so beneficial while, for ethanol 50%, the increase in temperature improved a lot the solvating power and gave a good TPC yield. Continuous extraction did not show any significant difference dependent on the use of one solvent or the other at 25 °C but the increase in temperature highly improved the extraction power of ethanol 50% which achieved the highest results in extractable TPC per gram of dry seeds. Water provided very good results with both batch and continuous extraction and at 25 °C the former led to a higher TPC content probably due to a weaker solvating power of water for the antioxidant compounds of interest at this temperature in continuous solvent flow conditions (at this specific rate of 1 mL/min). In batch conditions, the residence time is very long and solubility of solute, good stirring, high mass transfer and high contact surface area seem to be adequate conditions for reaching equilibrium concentration in at least 4 h. Water extraction in the optimum conditions in terms of cost and time (25 °C for 4 h) yielded twice the TPC obtained in the same conditions with ethanol 50% and only with a temperature increase (up to 60 °C), was it found that ethanol 50% provided comparable results to those obtained with water. An interesting observation is the totally different trend of extraction power: increasing the temperature and reducing the extraction time, water showed a decrease results reaching the best yield with the severest conditions (60 °C for 1 h), even if the yield was still lower than that obtained with water at room temperature. This could be explained by the fact that pure hot water could favour the solubility of polyphenols and other molecules with similar characteristics to polyphenols (such as anthocyanins, tannins, saponins and terpenoids [1]) lowering their extraction rate and generating interferences in the Folin-Ciocalteau analysis. The increase in temperature could also lead to instability and thermal degradation of antioxidant molecules, significantly reducing the detected TPC. Pure water selectivity towards target molecules (polyphenols) is high at low temperatures (25 °C) but decreases for higher values (60 °C) while its solvating power greatly increases. Ethanol, despite the lower solvating power, shows good selectivity with respect to polyphenols mainly at higher temperature [15]. Based on further performed tests, an increase in temperature of 56 °C (test at 4 °C and test at 60 °C) provides an improvement of 80% in yield of extracted TPC with ethanol 50% while the same increase in temperature leads to a drop of 26% in yield of extracted TPC with water.

There are very few previous studies regarding this topic. One that could be highlighted is from Fontoura et al. (2015) who performed extraction of polyphenols from *Sterculia apetala* bark and gave a value of TPC of 59.7 ± 13.9 GAE mg/g of sample using Ultrasound extraction (for 60 min and 24 h of maceration) in ethanol 20% [13] while the results obtained by the use of *Sterculia apetala* seeds in this experimental work provided a value of 4.33 ± 0.24 GAE mg/g of sample (results comparable to those obtained by Sha Li et al. [16]) using maceration in water at room temperature for 4 h.

### 2.4. Fatty Acid Content

Gas Chromatography allowed analysis of fatty acid content with retention times of peaks used to identify different fatty acids extracted from the samples and their area, the concentration. In *Sterculia apetala* seeds, the study of Herrera-Meza et al. identified the presence of different fatty acids such as palmitic, oleic, sterculic and malvalic acid [14].

**Figure 5:**
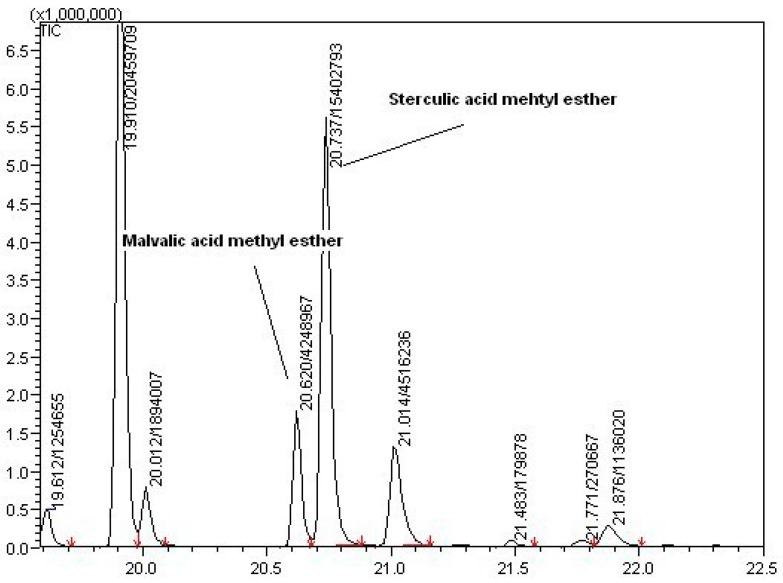
Spectrum of Gas Chromatography to detect the fatty acids present in Sterculia seeds with focus on malvalic and sterculic acids values of the area under the peak of the gas chromatogram. *Sterculia apetala* seeds are a good source of fatty acids and, in particular, palmitic acids between the others with a percent value of 39% on the total amount of fatty acids followed by oleic (23%), sterculic (20%) and malvalic (7%) (Herrera-Meza et al. found a composition of palmitic (19%), oleic (9.5%), sterculic (56%) and malvalic (1.3%) acids in roasted seeds of *Sterculia apetala* [14]).

The chromatogram in Figure 5 shows four main peaks. Palmitic acid is present in the largest amount in all the samples but it has been possible to detect the concentration of only palmitic and oleic acids, for lack of a reference curve to which to compare sterculic and malvalic acids. However, considering the molecular weight of these fatty acids and data from the literature, there is a high probability that the two not identified peaks would represent malvalic and sterculic acids.

## 3. Materials and Methods

### 3.1. Sample and Extracts Preparation

The natural samples are seeds of *Sterculia apetala* tree and they were collected in Mexico in different periods: January, February and May of 2015. For this reason, they show different features: bigger size, softer consistency and clearer peel colour for the less mature ones and smaller size, harder consistency and brown-black colour for the seeds collected at the beginning of the summer. A mix of all the three harvest variants was used for performing the solvent extractions. The cut seeds are considered as spherical particles with a diameter of 0.6 ± 0.1 cm and 0.8 ± 0.1 cm for dried and fresh seeds (humidity 74–82%) respectively and weight in the range 0.4–0.6 g. Extracts were prepared with 1 g of sample on 20 mL of 50% EtOH.

### 3.2. Materials and Reagents

Absolute ethanol (96% *v*/*v* partially denatured), Methanol, Anhydrous Sodium Carbonate and *n*-Hexane were purchased from Panreac, Barcelona, Spain. Potassium Hydroxide, Gallic Acid, Folin-Ciocalteau reagent, AAPH 2,2′-Azobis(2-methyl-propionamide) dihydrochloride, Phosphate Buffered Saline Tablets (PBS), Fluorescein Sodium Salt, Trolox^®^ (6-hydroxy-2,5,7,8-tetramethylchroman-2-carbonsaeure), Potassium Peroxidisulfate, ABTS (2,2′-Azino-bis(3-ethylbenzothiazoline-6-sulfonic acid diammonium salt) were acquired from Sigma-Aldrich, Madrid, Spain. Pure water and Distilled water (Millipore-Q system, Barcelona, Spain). 

### 3.3. Chemical Analysis

#### 3.3.1. Determination of Total Phenolic Content (TPC)

Total Phenolic Content was quantified with the use of the Folin-Ciocalteau method described by Singleton, Orthofer and Lamuela-Raventos using gallic acid as standard [18]. A 96-well microplate was used, adding with a multichannel micropipette, 20 µL of sample, 80 µL of Folin-reagent (0.62 M), 80 µL of 4% (*w*/*w*) saturated sodium carbonate and 80 µL of Milli-Q water to each well. The microplate was incubated in the dark at 25 °C and absorbance was measured after one hour at 765 nm using a Fluorimetrics Fluostar Omega (Perkin–Elmer, Paris, France). Results are expressed in mg Gallic Acid Equivalents (mg GAE) ± standard deviation based on the calibration curve y = 0.891x − 0.0365 with R^2^ = 0.9929. Each sample was analysed in triplicate.

#### 3.3.2. Determination of the Antioxidant Activity (ABTS Assay)

The modified experimental method used by Miller et al. (1996) [19] was followed using the radical ABTS*^•^*^+^ generated by mixing an ABTS solution 7 nM with a potassium persulfate solution of 2.45 mM and storing it in the dark for at least 16 h. The ABTS solution was prepared by diluting it in PBS until reaching an absorbance value between 0.7–1 at 734 nm (measured after a first reading) adding 200 µL to a microplate well. Using a microchannel micropipette, samples were added to the plate in addition to the ABTS solution and then stirred and incubated in the dark at 30 °C in the Fluorimetrics Fluostar Omega (Perkin–Elmer, Paris, France). Absorbance was measured at 734 nm after 10 min and antioxidant power is expressed in µmol Trolox Equivalent (TE)/g ± standard deviation by referring to a Trolox standard calibration curve y = 0.0025x + 0.0148 with R^2^ = 0.9963. Each sample was analysed in triplicate.

#### 3.3.3. Fatty Acid Content (Gas Chromatography)

Fatty acid content was determined by Gas Chromatography coupled to Flame Ionization Detector (GC-FID) (GC2025 Shimazdu, Kyoto, Japan) with an automatic injector (AOC20i) operating at a maximum of 260 °C equipped with a BPX-70 (SGE) capillary column (30 m × 0.25 mm i.d. × 0.25 μm f.d.). The injection volume was 1 μL, the carrier gas was helium at a flow of 1.14 mL/min and the mode of injection was split, ratio 20:1. The oven temperature program was: 60 °C (1 min) then slope of 6 °C/min until 260 °C (30 min). The detector operated at 280 °C with helium flow, 30 mL/min; hydrogen flow, 40 mL/min; and airflow 400 mL/min. Analysis were performed in duplicate. Extracts were introduced with a 1:10 dilution, previously filtered with 0.45 μm nylon filters. Obtention of fatty acids is described in Section 3.7.

### 3.4. Continuous Solid-Liquid Extraction

**Figure 6:**
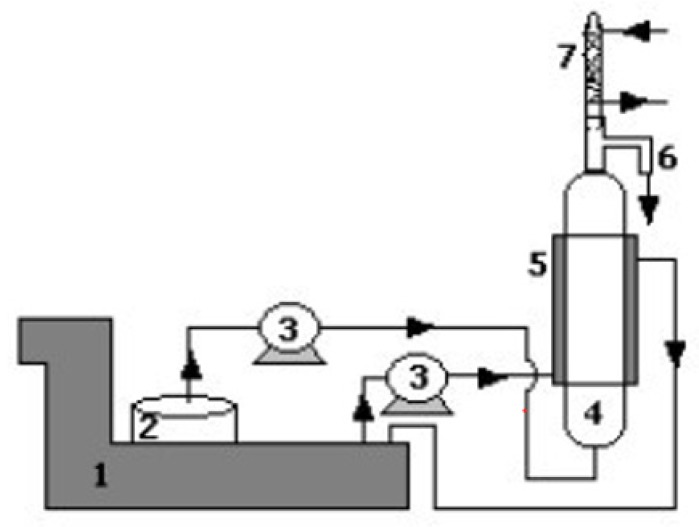
Schematic laboratory apparatus for continuous extraction: (1) water bath, (2) fresh solvent vessel, (3) peristaltic pumps, (4) extraction column, (5) column jacket, (6) extraction outlet, (7) condenser.

The experimental apparatus was built using a jacketed extraction column of 12 cm in height, 1 cm as internal diameter and 1.5 cm as total diameter with the inner space used as sample holder through which the extraction solvent (water or ethanol 50%) flowed as shown in Figure 6 [20]. A mixed sample of fresh and dry seeds in pieces (9 ± 0.2 g) was placed inside and blocked with glass wool at the bottom and at the top of the column to avoid sample entrainment.

The experimental temperature was controlled with the use of a water bath set to the selected temperature (25 °C or 60 °C) in which the flask (containing the fresh solvent) was placed. The fresh solvent and the water in the column jacket were pumped with the use of two peristaltic pumps. The solvent flow rate was 1 ± 0.2 mL/min and was maintained constant. The extract was taken from the top of the column and collected as samples of 1 mL at different time intervals with a total test duration of 6 h (the total extraction solvent was collected in a flask placed under the apparatus).

### 3.5. Determination of the Effective Diffusion Coefficients in the Continuous Extraction

The effective diffusion coefficients were determined for the continuous solid-liquid extraction. Assuming no change of the effective diffusivity with the solute concentration, the rate of diffusion can be described by the second Fick’s law (Equation (3) [17]) and, considering that particles have a spherical shape, the same equation can be written in spherical coordinates (Equation (4) [17]):

(3) $$\frac{DC_{A}}{Dt}=D_{\mathrm{AB}}\nabla^{2}c_{A}$$

(4) $$\frac{\partial C}{\partial t}=D_{eff}(\frac{1}{r^{2}}\frac{\partial }{\partial r}(r^{2}\frac{\partial C}{\partial r}))$$

where *r* is the diffusion radius, *C*_**A**_ the concentration of the compound **A**, *D*_**AB**_ the diffusion coefficient of the compound **A** into the solvent B and *D_eff_* the effective diffusion coefficients. The most common approach for calculating the *D_eff_* in a solid-liquid extraction is based on the experimental determination of kinetics [19]. The extraction is assumed to occur under the following conditions: the solid material can be considered as an assembly of spherical particles of the same size, the particle diameter corresponds to the average diameter of the sample, the diffusion of the solute and other compounds is carried out in parallel and there is no interaction between them, the diffusivity of the extracted solute is constant, the controlling stage of the process is the internal diffusion and the extracted polyphenols are considered as a single compound, equivalent to gallic acid [11,15]. The extension of the model to a packed bed inside the extraction column can be made on the assumption that all the particles behave in the same way [15]. In the case of a solid-liquid extraction in a continuous flow of a solvent, the diffusivity parameter could be determined by applying the second Fick’s law (assuming that *D_eff_* would be constant with the concentration) such as the expression proposed by Pinelo et al. (2006) (Equation (5)) [17]:

(5) $$Y=\frac{6}{\pi^{2}}\sum_{n=1}^{\infty }\frac{1}{n^{2}}e^{-(\frac{n^{2}\pi^{2}D_{eff}\cdot t}{r^{2}})}$$

with the following boundary conditions: *Y* = 0, *r* ± *R*, *t* ≥ 0; *Y* = 1, 0 < *r* < *R*, *t* = 0. *Y* is defined as the unextracted fraction of phenolic compounds from the matrix and is calculated as the ratio between the internal phenol concentration at a specific time t and the initial phenol concentration in the seed before the extraction. Estimating the effective diffusivity at large values of time (in order to avoid the effect of initial operational factors) where the Fick’s number is great and the model can be reduced to the first term (*n* = 1) with a small error [17]. The estimated area under the curve in the plot of TPC concentration against extraction time permits then to obtain the approximate value of the extracted phenolic compounds (mg GAE) at specific periods of time [17].

From these, the value of the unextracted fraction of polyphenols can be calculated as (Equation (6)) [20]:

(6) $$Y=\frac{mgGAE_{0}-mgGAE(t)}{mgGAE_{0}}$$

where *mgGAE*_0_ is the mass of polyphenols present at the initial conditions inside the seed matrix while *mgGAE*(*t*) is the measured total polyphenols extracted in the liquid solvent at the specific time t. The initial amount of TPC contained in the *Sterculia apetala* seeds (*mgGAE*_0_), in absence of a known estimated specific chemical composition, has been assumed to be equal to the maximum extractable amount from seeds obtained using the extraction technique with the highest extraction yield with a value of extracted polyphenols corresponding to 0.5% of the total weight of a seed (1 ± 0.2 g). For this reason, the initial total mass of polyphenols is assumed to be around 0.5% of the seed weight. Linearizing the Equation (5) and plotting the logarithm of the unextracted fraction against time, a straight line can be obtained and the slope term could be used to calculate the value of the effective diffusivity as shown in Equation (7) [17]:

(7) $$\ln Y=\ln(\frac{6}{\pi^{2}})-\frac{\pi^{2}D_{eff}}{r^{2}}t$$

This procedure has been followed in several works to evaluate the diffusivity values from experimental data (Palumbo et al., 1997 [21], Schwartzberg and Chao, 1982 [22]) [17]. In the followed procedure, the system is considered as a packed bed of seeds assumed as spherical particles with a radius equal to 2.5 ± 0.1 mm with a total weight of 9 g. Once fitted to the experimental data, the obtained intercept is different from the term $\ln(\frac{6}{\pi^{2}})$ of Equation (7) and the adjusted kinetic equation to these experimental data assumes the form of Equation (8)

(8) $$\ln Y=B-\frac{\pi^{2}D_{eff}}{r^{2}}t$$

where *B* is the obtained intercept from the straight line which better approximates the data trend. The slope term permits calculation of the value of the effective diffusion coefficient by linear regression of the experimental data

### 3.6. Batch Solvent Extraction

Besides continuous solid-liquid extraction, maceration under stirring was performed placing the sample (ground to a powder to have a very high surface contact area) in a solvent for a determined period of time, in order to reach a high concentration of the extracted compounds in the liquid. Millipore Water and an aqueous solution of ethanol (concentration of 50%) were used. 20 mL of different solvents were placed in plastic cylindrical vials containing 1 g of ground seeds. The vials were placed on a magnetic stirrer/heater and the extraction was performed at 25 °C for 4 h or at 60 °C for 1 h (to avoid an excessive thermal degradation of the sample).

### 3.7. Fatty Acid Extraction

The precise weight of empty plastic vials was measured using an analytical balance with high precision (5 decimals) and then, previously frozen and crushed seeds were placed into n-hexane to perform the extraction, following a proportion of 1 g/6 mL of solvent. The vials were covered with pitted parafilm (permeable) and left for at least five days to allow complete extraction of fatty acids by hexane, followed by its total evaporation. Once fatty acids were extracted and deposited on the bottom of the vial, seeds were removed and vials weighed again to compare with the weight of the empty ones and to measure the amount of the extracted fatty acids. Fatty acid methyl esters (s) were recovered using methodology described in literature with modifications [23,24]: 0.1 g of extract was esterified by saponification using 200 µL of KOH solution in methanol (2 M) and fatty acids were recovered by adding to 2 mL of *n*-hexane. Finally, samples were centrifuged at 2000 g for 10 min and the supernatant was kept at −80 °C until analysis.

## 4. Conclusions

Different methods for extracting antioxidant compounds from seeds of *Sterculia apetala* have been studied. Batch solvent extraction under continuous stirring is likely to be the most used, cheap and easy method for the extraction of antioxidant compounds from natural raw materials. An increase in temperature up to 60 °C increases the diffusion rate and achieves a good solvating power. Water has consistently led to better extraction than ethanol 50%. Considering all aspects including convenience, batch extraction with continuous stirring at room temperature using water as solvent and seeds ground to a powder are the optimal conditions to obtain the best yield of antioxidant compounds from *Sterculia apetala* seeds. Moreover, a continuous process requires the use of an extraction apparatus made of different units involving the presence of peristaltic pumps, an extraction column and a solvent bath. There is limited literature research on this kind of plant and previously studies were related to the investigation of the antioxidant properties of samples of bark or leaves of different varieties of Sterculia. Comparing our values with the literature, it is clear that seeds contain less antioxidant compounds than other plant parts but also the kind of extraction technique, the solvent used and the analysis methods are important factors affecting the comparison. *Sterculia apetala* seeds seem not to be a major source of antioxidant compounds compared to many other plants [16].

## Figures and Tables

**Table 1:** Direct comparison of using water and ethanol 50% at different temperature in a continuous solid-liquid extraction in terms of TPC and antioxidant activity of the extracted samples.

Extraction Solvent	Temperature (°C)	Time (h)	TPC (mg GAE/g of Dry Sample)	ABTS (µmol/g of Dry Sample)
Water	25	6	2.57 ± 0.13	50 ± 0.4
	60	6	4.13 ± 0.14	76.3 ± 0.6
Ethanol 50%	25	6	2.37 ± 0.85	43.1 ± 0.8
	60	6	4.72 ± 1.34	56.2 ± 0.7

**Table 2:** Obtained values of the effective diffusion coefficient of polyphenols extracted by *Sterculia apetala* seeds in a continuous extraction with water and ethanol 50% at 25 °C and 60 °C.

Solvent	Temperature (°C)	Slope × 10^−5^	*Deff* (m^2^/s)	Regression Coefficient
Water	25	−1.82	1.15 × 10^−11^	0.982
	60	−3.31	2.10 × 10^−11^	0.996
Ethanol 50%	25	−2.17	3.71 × 10^−11^	0.997
	60	−5.86	1.38 × 10^−11^	0.998

**Table 3:** Comparison between the use of batch or continuous solid-liquid extraction in TPC obtainment form *Sterculia apetala* seeds samples varying in particle size and extraction period.

Extraction Solvent	Extraction Methodology	Particle Size Diameter (mm)	Temperature (°C)	Time (h)	TPC (mg GAE/g of Dry Sample)
Water	Batch extraction (Stirring conditions)	≤0.1	25	4	4.33 ± 0.24
		≤0.1	60	1	3.7 ± 0.42
	Continuous extraction	7 ± 1	25	6	2.57 ± 0.13
		7 ± 1	60	6	4.13 ± 0.14
Ethanol 50%	Batch extraction (Stirring conditions)	≤0.1	25	4	2.28 ± 0.31
		≤0.1	60	1	3.08 ± 0.12
	Continuous extraction	7 ± 1	25	6	2.37 ± 0.85
		7 ± 1	60	6	4.72 ± 1.34
